# Diagnosis and Management of Hypertensive Heart Disease: Incorporating 2023 European Society of Hypertension and 2024 European Society of Cardiology Guideline Updates

**DOI:** 10.3390/jcdd12020046

**Published:** 2025-01-26

**Authors:** Brian Xiangzhi Wang

**Affiliations:** Department of Medicine, Jersey General Hospital, St. Helier, Jersey JE1 3QS, UK; brian.wang15@imperial.ac.uk

**Keywords:** hypertension, arrhythmia, heart failure, cardiomyopathy, heart disease

## Abstract

Hypertensive heart disease (HHD) continues to be a leading cause of cardiovascular morbidity and mortality worldwide, necessitating the evolution of evidence-based management strategies. This literature review examines the most recent updates from the 2023 and 2024 hypertension guidelines issued by the European Society of Hypertension (ESH) and the European Society of Cardiology (ESC). These guidelines are compared with previous key recommendations, such as the 2017 American College of Cardiology/American Heart Association guidelines and the 2018 ESC/ESH guidelines. The updated recommendations reflect a paradigm shift in the approach to hypertension diagnosis and management, including a stricter systolic blood pressure (BP) target of 120–129 mmHg, which underscores the importance of early and precise BP control. The difference between the classification of “elevated BP” and hypertension in the ESC versus ESH guidelines, particularly, regarding their implications for early detection and prevention of HHD, are critically examined, highlighting areas of clinical and academic debate. The introduction of a new “elevated BP” category (120–139/70–89 mmHg) highlights a proactive strategy aimed at identifying at-risk individuals earlier in the disease course to prevent progression to HHD. Additionally, the divergent roles of hypertension-mediated organ damage (HMOD), including HHD, in risk stratification as recommended by the ESC and ESH are discussed, emphasising their significance in tailoring management approaches. For patients with resistant hypertension, the 2023 and 2024 updates also endorse innovative therapies, such as renal denervation, an interventional procedure that has demonstrated significant promise in managing treatment-resistant cases. This review synthesises these updates, focusing on their implications for clinical practice in diagnosing and managing HHD. By emphasising aggressive intervention and the integration of novel treatment modalities, the review aims to bridge existing gaps in earlier approaches to hypertension management. The critical evaluation of guideline discrepancies and evolving evidence seeks to provide clinicians with a nuanced understanding to optimise outcomes for patients with HHD, particularly considering emerging therapeutic possibilities and more stringent BP control targets.

## 1. Introduction

Hypertensive heart disease (HHD) refers to a complex spectrum of cardiovascular conditions induced by sustained high blood pressure (BP), which includes left ventricular hypertrophy (LVH), heart failure, ischemic heart disease, and arrhythmias. The pathogenesis of HHD arises from chronic exposure to elevated BP, leading to structural and functional cardiac alterations. Globally, hypertension affects over 1.28 billion people, making it one of the most significant risk factors for cardiovascular morbidity and mortality [1,2]. Many patients remain asymptomatic until advanced cardiac damage has occurred, highlighting the critical need for early diagnosis and effective management of hypertension to prevent HHD progression and reduce associated cardiovascular risks.

Over the past decade, our understanding and management of hypertension and HHD have advanced considerably, with clinical guidelines evolving to reflect emerging evidence. Foundational guidelines, such as those issued by the American College of Cardiology (ACC) and the American Heart Association (AHA) in 2017, as well as the European Society of Cardiology (ESC) and the European Society of Hypertension (ESH) in 2018, recommended a target BP of <130/80 mmHg for most individuals [3,4]. These recommendations underscored the importance of early hypertension diagnosis, risk stratification, and lifestyle modification in reducing HHD-related morbidity and mortality. However, subsequent landmark trials such as the STEP (Strategy of Blood Pressure Intervention in the Elderly Hypertensive Patients) study and the SPRINT (Systolic Blood Pressure Intervention Trial) have provided compelling evidence that more aggressive BP control may yield additional cardiovascular benefits, thereby prompting a reconsideration of BP targets [5,6].

The recent 2023 ESH and 2024 ESC hypertension guidelines reflect a paradigm shift towards more intensive BP control, lower BP thresholds, and broader therapeutic strategies [2,7]. The 2024 ESC guidelines now advocate for a lower systolic BP target range of 120–129 mmHg, underscoring a more proactive approach to BP management. Additionally, the guidelines introduce an “elevated BP” category (120–139/70–89 mmHg), which emphasises the importance of early identification and intervention for individuals at risk of cardiovascular disease (CVD) [2]. This lower threshold is particularly relevant for high-risk groups such as those with diabetes or chronic kidney disease (CKD), where early and intensive BP control has been shown to reduce cardiovascular events significantly.

The guidelines also highlight the importance of hypertension-mediated organ damage (HMOD), including HHD, in risk stratification, where differences in ESC and ESH recommendations offer further opportunities for discussion and refinement. For example, ESC guidelines place a heightened emphasis on HMOD as a critical component of cardiovascular risk assessment, influencing the prioritisation of treatment intensity and monitoring strategies.

Another notable update in the 2024 ESC guidelines is the recommendation of renal denervation for patients with resistant hypertension, a condition historically challenging to manage with pharmacologic therapy alone. Resistant hypertension, defined by persistently elevated BP despite the use of three or more antihypertensive agents, has been linked to a higher risk of adverse cardiovascular outcomes in HHD patients [8,9]. The inclusion of renal denervation as a treatment option signifies an important advancement, offering clinicians an alternative therapeutic approach for this subset of patients and reflecting a growing recognition of the need for diverse interventions in hypertension management.

This manuscript provides a comprehensive review of the recent 2023 and 2024 guideline updates, focusing on their implications for the diagnosis and management of HHD. By examining these new recommendations and comparing them with previous guidelines, we aim to underscore the clinical significance of incorporating the latest evidence-based practices. The shift towards earlier intervention, stricter BP targets, and novel therapeutic options marks a pivotal change in HHD management, with the potential to substantially enhance patient outcomes and reduce the global burden of hypertension-related CVD.

## 2. Pathophysiology of HHD

HHD is driven by chronic elevated BP that results in multiple structural and functional changes in both the heart and vasculature. The pathophysiology of HHD involves several interrelated mechanisms, including LVH, vascular remodelling, myocardial fibrosis, and impairments in both diastolic and systolic function. Collectively, these alterations elevate the risk of heart failure, arrhythmias, and ischemic heart disease, marking HHD as a significant cardiovascular disorder with complex underlying mechanisms. The 2023 and 2024 guidelines underscore the need to address these pathophysiological features through early and precise risk stratification, emphasising the use of HMOD, including HHD, to guide treatment intensity and improve outcomes.

### 2.1. LVH

LVH is considered the hallmark of HHD. It is characterised by thickening of the myocardium in response to chronic pressure overload [10]. Prolonged hypertension increases systemic vascular resistance, requiring the left ventricle to exert greater force to eject blood, leading to adaptive hypertrophy of myocardial cells [11]. Initially, this hypertrophic response is beneficial, helping the heart maintain cardiac output despite the increased workload. However, over time, persistent hypertrophy becomes maladaptive, leading to structural and functional impairments [12].

Histopathologically, LVH is characterised by the enlargement of cardiomyocytes, increased interstitial fibrosis with collagen deposition, and impaired coronary microcirculation [13,14]. These changes contribute to diastolic dysfunction, as the stiffened ventricle struggles to relax and fill during diastole. In advanced stages, LVH may also lead to systolic dysfunction, where the heart’s ability to contract effectively is compromised. The 2024 ESC guidelines highlight the importance of early recognition and management of LVH as it is predictive of future cardiovascular events, including heart failure and arrhythmias such as atrial fibrillation (AF) [2]. The guidelines advocate for incorporating LVH as a marker of HMOC in risk stratification while highlighting differences from the ESH’s less specific approach to HMOD in overall risk assessment.

### 2.2. Vascular Remodelling and Arterial Stiffness

Vascular remodelling and arterial stiffness are key components in the progression of HHD. Chronic hypertension causes structural changes in the arterial walls, leading to thickening and loss of elasticity, known as arterial stiffness. Sustained high BP generates mechanical stress on the arterial walls, resulting in vascular smooth muscle proliferation and increased deposition of extracellular matrix proteins [15]. This process leads to reduced arterial compliance and a heightened systolic BP, which increases the workload on the left ventricle.

The stiffened arteries impair the vasculature’s ability to buffer the pulsatile blood flow, allowing higher systolic pressures to reach the microvasculature of critical organs, including the heart [16]. This effect can worsen myocardial ischemia and accelerate the progression of LVH and heart failure. The 2024 ESC guidelines place greater emphasis on arterial stiffness as an important component of HMOD, integrating it into cardiovascular risk stratification more explicitly than prior ESC/ESH guidelines, and distinctively from the ESH’s approach. Additionally, endothelial dysfunction often accompanies arterial stiffness in hypertensive patients, reducing nitric oxide availability and further impairing vascular relaxation [17]. This dysfunction is a precursor to atherosclerosis and contributes to the development of coronary artery disease (CAD) in hypertensive patients, further complicating HHD management.

### 2.3. Myocardial Fibrosis

Myocardial fibrosis represents a critical pathophysiological feature of advanced HHD. As the heart adapts to chronic pressure overload, there is an increased deposition of collagen and other extracellular matrix components in the myocardial interstitium [18]. This process disrupts the normal architecture of cardiac tissue, reducing its elasticity and impairing both diastolic and, eventually, systolic function. Stiffened myocardial tissue hampers diastolic filling, while extensive fibrosis in advanced stages can also affect systolic contraction.

A primary driver of myocardial fibrosis is the activation of the renin–angiotensin–aldosterone system (RAAS), which plays a pivotal role in the pathogenesis of HHD. Elevated levels of angiotensin II and aldosterone stimulate fibroblasts, leading to increased collagen synthesis and deposition [19]. The 2023 and 2024 guidelines emphasise the critical role of RAAS inhibition as a therapeutic strategy to reverse fibrosis and mitigate adverse remodelling. Notably, the ESC guidelines more prominently highlight these interventions within their framework for preventing HMOD progression, whereas ESH focuses on broader BP-lowering strategies [2,7].

### 2.4. Heart Failure

HHD is a leading cause of heart failure, particularly heart failure with preserved ejection fraction (HFpEF). In the presence of LVH and myocardial fibrosis, the left ventricle’s diastolic function is compromised, leading to increased left atrial pressure and subsequent pulmonary congestion. Patients with HFpEF commonly present with symptoms such as dyspnoea, exercise intolerance, and fluid retention, although they maintain normal systolic function in the early stages.

Over time, if hypertension remains untreated or inadequately managed, systolic dysfunction may also develop, progressing to heart failure with reduced ejection fraction (HFrEF). The transition from hypertension to overt heart failure is often influenced by comorbidities such as CAD, which reduces myocardial perfusion, and CKD, which further exacerbates volume overload [20,21]. The 2024 ESC guidelines underscore the importance of aggressive hypertension control and early identification of HMOD, including diastolic dysfunction, to prevent progression to heart failure. This contrasts with the ESH’s more generalised recommendations for early BP management without as specific an emphasis on HMOD.

### 2.5. Arrhythmia Risk

HHD predisposes patients to arrhythmias, with AF being particularly prevalent [22]. Diastolic dysfunction and LVH cause elevated left atrial pressures and atrial dilation, creating a substrate for the development of AF. In patients with hypertension and HHD, AF can further compromise cardiac output and increase the risk of thromboembolic events, such as stroke. The 2023 and 2024 guidelines highlight the importance of strict BP control in preventing AF-related complications. Notably, the ESC guidelines provide more specific recommendations for integrating arrhythmia management into overall HHD care compared to the ESH’s broader emphasis on BP targets [2,7].

### 2.6. Neurohormonal Activation

In HHD, neurohormonal activation plays a significant role in driving the progression of cardiac and vascular pathology. Chronic hypertension leads to the persistent activation of the RAAS and the sympathetic nervous system [23,24]. These systems promote vasoconstriction, sodium retention, and increased blood volume, which collectively contribute to elevated BP and heightened cardiac workload. Sustained neurohormonal activation also induces adverse myocardial remodelling and fibrosis, and exacerbates LVH, furthering the structural damage and functional impairments in HHD [25].

Additionally, elevated sympathetic activity can precipitate arrhythmias, accelerate heart rate, and increase myocardial oxygen demand, all of which worsen cardiac function [26]. The guidelines uniformly recommend targeting these pathways with therapies such as beta-blockers, RAAS inhibitors, and mineralocorticoid receptor antagonists. However, the ESC provides more detailed guidance on using these therapies to address HMOD, distinguishing its recommendations from the ESH’s less targeted approach.

## 3. Clinical Presentation and Diagnosis

HHD often progresses silently over the years, with clinical manifestations that typically arise only in advanced stages. The presentation varies based on the specific cardiac complications that develop, including LVH, heart failure, ischemic heart disease, and arrhythmias. Early identification of these manifestations is essential for timely intervention, as significant cardiac damage may already be underway by the time symptoms become apparent.

### 3.1. Clinical Presentation of HHD

The clinical course of HHD is insidious, with many patients remaining asymptomatic for extended periods. When symptoms do appear, they are often subtle and may not immediately point to cardiac issues, leading to delays in diagnosis. The ESC guidelines emphasise leveraging advanced diagnostic tools, while the ESH guidelines favour integrating simpler clinical parameters to stratify risk in asymptomatic patients. The clinical presentation of HHD can be broadly categorised as follows:

#### 3.1.1. Asymptomatic Stage

In the early stages, HHD may be detected only incidentally through routine BP measurements or cardiovascular risk assessments. Patients with LVH, for instance, may not experience any symptoms, and the hypertrophy is identifiable only through imaging [27]. This silent progression underscores the importance of regular screenings and early hypertension management to identify HHD before clinical symptoms arise. The 2023 and 2024 ESC guidelines advocate for using echocardiographic and MRI-based detection of subclinical HMOD to enhance early diagnosis, contrasting with the ESH’s focus on broader population-based strategies.

#### 3.1.2. Symptoms of Heart Failure

As HHD advances, diastolic dysfunction can develop, leading to HFpEF. Common symptoms include exertional dyspnoea, orthopnoea, and paroxysmal nocturnal dyspnoea. Fatigue, peripheral oedema, and reduced exercise tolerance are also common, indicating progression to symptomatic heart failure. In some cases, left ventricular systolic dysfunction may emerge, resulting in HFrEF, accompanied by more severe symptoms of cardiac decompensation. The ESC guidelines highlight the need for identifying subclinical diastolic dysfunction through advanced imaging, emphasising its predictive value in preventing overt HF.

#### 3.1.3. Chest Pain and Ischemic Symptoms

LVH increases myocardial oxygen demand and, together with coronary microvascular dysfunction, raises the risk of ischemic heart disease in hypertensive patients. These patients may experience angina-like chest pain during exertion or even silent myocardial ischemia, especially in the presence of concurrent CAD. Recognising these symptoms in hypertensive individuals is crucial to managing ischemic risks and preventing further complications. Both guidelines recommend integrating coronary artery assessment into routine HHD evaluation, particularly in high-risk populations, to mitigate ischaemic complications.

#### 3.1.4. Arrhythmias

AF is a common complication in HHD, especially in individuals with long-standing hypertension, LVH, or left atrial enlargement [28]. AF presents with palpitations, light-headedness, and, in some cases, syncope, particularly if ventricular rates are poorly controlled. The presence of AF in hypertensive patients further elevates the risk of thromboembolic events such as stroke and can exacerbate heart failure symptoms [29]. The guidelines highlight differences in their approach to managing arrhythmias; the ESC emphasises BP control with targeted rhythm management strategies, while the ESH provides more generalised BP-lowering recommendations.

### 3.2. Diagnostic Evaluation

Prompt and comprehensive evaluation of HHD is essential to prevent irreversible cardiac damage. The diagnostic approach combines clinical assessment, imaging, and laboratory tests to identify the structural and functional impacts of hypertension on the heart and guide therapeutic decisions.

#### 3.2.1. History and Physical Examination

A detailed medical history and physical examination are foundational in assessing HHD:

- BP monitoring: Accurate BP measurement remains a cornerstone of HHD diagnosis. The 2023 and 2024 guidelines recommend supplementing office BP measurements with 24 h ambulatory BP monitoring (ABPM) and home BP monitoring (HBPM) to detect masked or white-coat hypertension [2,7]. These methods provide a more comprehensive view of BP patterns and can reveal otherwise unrecognised hypertension, critical for cardiovascular risk stratification;
- Assessment of target organ damage: A physical examination should focus on identifying evidence of target organ damage, such as hypertensive retinopathy, carotid, or abdominal bruits (indicating vascular disease), and signs of LVH, such as a displaced or sustained apical impulse. The ESC guidelines place greater emphasis on clinical markers of HMOD during routine physical evaluations compared to the ESH.

#### 3.2.2. Diagnostic Tests

A range of diagnostic tools is available to evaluate the impact of hypertension on cardiac structure and function:

- Echocardiography: This is the gold standard for assessing left ventricular hypertrophy, diastolic dysfunction, and left atrial enlargement [30]. Echocardiography provides detailed information on heart structure and function, measuring parameters such as ventricular wall thickness, chamber sizes, and ejection fraction. Advanced echocardiographic techniques, like strain imaging, can detect subclinical myocardial dysfunction, enabling earlier intervention [31]. The 2023 and 2024 guidelines reinforce the importance of echocardiography in routine HHD evaluation [2,7];
- Electrocardiography (ECG): Though less sensitive than echocardiography, ECG is a valuable tool for identifying LVH and arrhythmias. Specific criteria, such as the Sokolow–Lyon index and Cornell voltage criteria, help detect LVH, while atrial enlargement and arrhythmias, including AF, are also identifiable [32]. ECG remains a key screening tool, particularly in primary care settings;
- Cardiac magnetic resonance imaging (MRI): cardiac MRI is the most accurate method for quantifying myocardial mass and detecting myocardial fibrosis, offering superior detail compared to echocardiography [33]. MRI is particularly valuable when echocardiographic findings are inconclusive or when fibrosis needs to be quantified to assess arrhythmia risk or progression of HHD. The 2023 and 2024 guidelines underscore MRI’s role in advanced diagnostic evaluations for HHD;
- Biomarkers: Biomarkers such as B-type natriuretic peptide (BNP) or NT-proBNP are useful in assessing myocardial stress and heart failure [34]. Elevated levels suggest increased myocardial wall stress and are indicative of heart failure, particularly HFpEF. These biomarkers support diagnostic evaluations and help guide further investigations in symptomatic patients;
- Laboratory Tests: Routine laboratory evaluations for suspected HHD include renal function tests, lipid profiles, and HbA1c to screen for diabetes and dyslipidaemia, both of which are common comorbidities that exacerbate cardiovascular risk. Comprehensive lab work helps identify factors that may worsen HHD and guides risk management strategies [35,36].

The ESC guidelines emphasise a multidisciplinary approach, integrating laboratory, imaging, and clinical findings for optimal HHF management, while the ESH promotes cost-effective and widely accessible diagnostic tools.

## 4. Risk Stratification and Prognosis

Accurate risk stratification is essential in HHD to predict outcomes, tailor treatment strategies, and reduce the likelihood of cardiovascular events such as heart failure, myocardial infarction, and stroke. The 2023 and 2024 hypertension guidelines place a renewed emphasis on integrating risk stratification into routine care to optimise treatment strategies, leveraging both traditional and advanced diagnostic tools. Both guidelines emphasise individualising treatment by assessing each patient’s cardiovascular risk based on various factors, including the severity of hypertension, comorbid conditions, target organ damage, and the presence of other risk factors like diabetes or CKD.

### 4.1. Cardiovascular Risk Scores

Cardiovascular risk scores remain foundational in stratifying patients based on their likelihood of adverse outcomes (Table 1). The SCORE2 algorithm, endorsed by the 2023 ESH and 2024 ESC guidelines, estimates 10-year risks for fatal and non-fatal cardiovascular events [37]. Similarly, the ACC/AHA ASCVD Risk Calculator provides robust predictions for American cohorts [38]. Both tools consider age, sex, cholesterol levels, smoking status, and systolic BP, but SCORE2 accounts for regional differences in cardiovascular risk within Europe, making it particularly suited for use in European populations.

The 2024 ESC guidelines reaffirm the role of SCORE2 in guiding treatment intensity [39]. Patients classified as high or very high risk—those with existing CVD, CKD, or diabetes—require aggressive BP control, with recommended systolic BP targets of 120–129 mmHg. A meta-analysis published in The Lancet demonstrated that achieving this tighter BP control with a 10 mmHg reduction in BP reduced the risk of major CVD events by 20%, consistent with the findings from the SPRINT trial [5,40]. This evidence underscores the importance of early and sustained risk stratification in managing hypertensive patients.

### 4.2. LVH and Its Prognostic Implications

LVH significantly worsens the prognosis of HHD. Studies have consistently shown that LVH, identifiable via echocardiography or ECG, correlates with increased risks of arrhythmias, heart failure, and sudden cardiac death [41,42]. The 2024 ESC guidelines stress the early detection of LVH as a critical component of initial risk stratification [2]. Advances in cardiac imaging, particularly cardiac MRI, now allow for the detection of myocardial fibrosis, a prognostic marker of advanced disease [43]. These updated guidelines underscore the need for intensified BP management and targeted therapies to address structural cardiac changes.

### 4.3. Prognosis in Heart Failure and AF

Heart failure, especially HFpEF, is a frequent complication of HHD with a poor prognosis. HFpEF predominantly affects older adults with long-standing hypertension and LVH. A 2023 study described HfpEF as a condition associated with elevated hospitalisation and mortality rates, particularly in patients with uncontrolled hypertension. Effective BP management was shown to mitigate these risks, reinforcing the importance of early and aggressive interventions [44]. The 2024 ESC guidelines emphasise systolic BP targets below 130 mmHg to reduce the progression to heart failure, particularly in patients with LVH and hypertension-induced cardiac remodelling. This aligns with growing evidence supporting aggressive BP management to improve outcomes.

Older adults are disproportionately affected by HFpEF. A study by Yang et al. (2022) in Clinical Cardiology highlighted that patients with HFpEF and poorly controlled hypertension have a 50% higher risk of hospitalisation and a 30% increased mortality rate compared to those with optimised BP control [45]. The 2024 ESC guidelines recommend early and sustained BP control, with a systolic BP target of less than 130 mmHg, to reduce the progression to HFpEF [2].

AF, another common complication of HHD, is both a marker of advanced disease and a contributor to adverse outcomes. AF increases the risk of thromboembolic events, including stroke, as well as worsening heart failure. Both the ESC and ESH guidelines recommend comprehensive AF management, integrating anticoagulation, rhythm or rate control, and aggressive BP optimisation. A study by Trullàs et al. (2023) in the European Journal of Internal Medicine reported that hypertensive patients with AF are 70% more likely to experience ischemic stroke than those without AF [46]. The latest guidelines emphasise comprehensive management, including anticoagulation, rhythm or rate control, and BP optimisation, to mitigate these risks [2,7].

### 4.4. Impact of Comorbidities on Prognosis

Comorbid conditions such as diabetes, CKD, and dyslipidaemia exacerbate the prognosis of HHD by accelerating vascular and myocardial damage. The presence of these comorbidities necessitates more aggressive BP management, with recent evidence supporting tighter targets of 120–129 mmHg for systolic BP.

Hypertension exacerbates CKD progression, increasing the risk of end-stage renal disease (ESRD). Conversely, CKD complicates BP control through mechanisms such as sodium retention and heightened RAAS activation. A study by Cushman et al. (2016) published in the American Journal of Nephrology demonstrated that maintaining a systolic BP below 130 mmHg in patients with CKD not only reduced cardiovascular events but also slowed the progression of kidney disease [47]. The study underscores the importance of aggressive BP management in this population to mitigate risks. The 2024 ESC guidelines highlight the use of RAAS inhibitors, such as ACE inhibitors or ARBs, for their dual cardio–renal protective effects. Evidence from a meta-analysis by Abu-Hantash et al. (2024) in *Medicina* supports these findings, showing improved outcomes in CKD patients receiving RAAS blockade therapy [48].

Hypertension and diabetes often coexist, compounding cardiovascular risks. The combination accelerates vascular damage, increasing the risk of myocardial infarction, stroke, and heart failure. A study by Ueki et al. (2023) in the Journal of Diabetes Investigation reported that tight glycaemic control in diabetic hypertensive patients significantly reduces cardiovascular complications [49]. The integration of advanced glucose-lowering therapies, such as SGLT2 inhibitors, in hypertensive diabetic patients has shown reductions in BP and cardiovascular mortality, as outlined in a 2024 review in Cardiovascular Diabetology [50].

Dyslipidaemia, commonly present in patients with hypertension, accelerates atherosclerosis and increases cardiovascular risk. Its management is critical in hypertensive patients. A 2023 study in the International Journal of Cardiology found that lipid-lowering therapies combined with aggressive BP management reduced major cardiovascular events by 32% in hypertensive patients with dyslipidaemia [50]. This aligns with both the ESH and ESC guidelines, which advocate combined lipid and BP management for comprehensive cardiovascular risk reduction.

### 4.5. HMOD in Risk Stratification

HMOD, including HHD, plays a central role in cardiovascular risk stratification across the ESC and ESH guidelines. However, notable differences exist in how each guideline incorporates HMOD into clinical decision-making. The ESC guidelines prioritise advanced imaging modalities, such as cardiac MRI, for detecting subclinical myocardial fibrosis, which offers superior prognostic value in assessing HHD progression. In contrast, the ESH guidelines rely on more accessible techniques, such as echocardiography and ECG, to identify LVH and other structural abnormalities.

These distinctions affect the stratification of patients into moderate-, high-, and very high-risk categories. For example, the ESC guidelines often classify patients with evidence of subclinical HMOD, such as myocardial fibrosis, into higher-risk groups, advocating for intensified treatment. The ESH guidelines, while acknowledging the prognostic value of HMOD, adopt a more traditional approach, particularly in settings with limited access to advanced imaging technologies. This variability underscores the need to balance resource availability with optimal patient care when integrating HMOD into risk assessment frameworks.

## 5. Management of HHD

Effective management of HHD aims to control BP, prevent target organ damage, and reduce the risk of complications such as heart failure and AF. The 2023 and 2024 hypertension guidelines emphasise a holistic, multi-faceted approach, combining lifestyle modifications, pharmacological therapy, and, in selected cases, interventional procedures [2,7]. Recent evidence supports not only reducing BP but also reversing or halting structural and functional cardiac changes caused by chronic hypertension.

### 5.1. Classification of “Elevated BP” vs. Hypertension in the ESC vs. ESH

An important point of contention in ESC and ESH guidelines is the classification of “elevated BP” and its implications for hypertension diagnosis and management. The ESC guidelines define “elevated BP” as a systolic BP of 120–129 mmHg, advocating for intensive lifestyle interventions to prevent progression to overt hypertension. In contrast, the ESH guidelines classify hypertension beginning at systolic BP ≥ 140 mmHg or diastolic BP ≥ 90 mmHg, reserving the “elevated BP” category for a more limited range of prehypertensive states. This divergence has clinical implications, as the ESC approach emphasises pre-emptive measures, potentially reducing the risk of HHD development through early lifestyle changes. The ESH definition, however, focuses on identifying patients who require immediate pharmacological treatment, particularly in high-risk cohorts.

**Figure 1:**
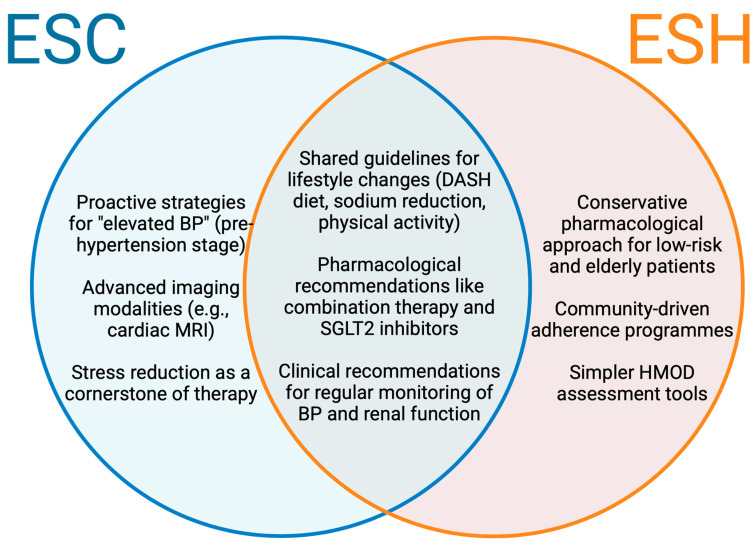
Prevention strategies across ESC and ESH guidelines. Venn diagram displaying the evidence-based interventions endorsed uniquely or heavily emphasised in the ESC guidelines (**left**), ESH guidelines (**right**), and both guidelines (**centre**) for HHD prevention. BP, blood pressure; ESC, European Society of Cardiology; ESH, European Society of Hypertension; HMOD, Hypertension-mediated organ damage.

These differences highlight the importance of tailored strategies, as the ESC’s emphasis on prevention aligns with evidence suggesting that early lifestyle modification can reverse preclinical cardiac remodelling, while the ESH’s threshold ensures resource allocation toward those at higher immediate risk (Figure 1).

### 5.2. Non-Pharmacological Management

Preventing the progression of hypertension-mediated organ damage, including HHD, requires timely interventions targeting elevated BP. The ESC guidelines underscore the importance of early identification and management of prehypertensive states to prevent target organ damage, promoting the use of the “elevated BP” as a precursor to hypertension. Lifestyle modifications, such as dietary interventions, physical activity, and stress management, are pivotal in this stage. Evidence suggests that sustained adherence to these measures reduces the risk of LVH and arterial stiffness, two hallmark features of HHD. The ESH guidelines, while more conservative in defining prehypertensive states, reinforce the importance of aggressive treatment for individuals already meeting hypertensive thresholds to halt disease progression.

Key Lifestyle Interventions include:

1. Dietary modifications:

Dietary patterns such as the DASH diet (Dietary Approaches to Stop Hypertension) have proven effective in reducing BP. This approach emphasises fruits, vegetables, whole grains, and low-fat dairy while minimising sodium, red meats, and added sugars. A review by Khanji et al. (2023) highlighted the DASH diet’s efficacy, including systolic BP reductions of 5–10 mmHg [51]. Additionally, potassium supplementation has been shown to lower BP, particularly in high-sodium consumers;

2. Sodium restriction:

Reducing sodium intake to less than 2.3 g per day significantly lowers BP [52]. A systematic review by Amirkhani et al. (2023) observed a direct correlation between sodium reduction and cardiovascular benefits [53]. The findings underscore stricter limits for patients with cardiovascular complications;

3. Physical activity:

Aerobic exercise, such as walking or jogging for at least 150 min weekly, can lower systolic BP by 5–7 mmHg [54,55]. The efficacy of exercise training was supported by a study that compared Western and Chinese exercise modalities in hypertensive patients, showing consistent BP reductions [56];

4. Weight reduction:

Maintaining a healthy body weight is essential for managing hypertension. Research by Slonovschi et al. (2023) indicates that weight loss interventions not only lower BP but also improve overall cardiovascular risk factors [57];

5. Smoking cessation and alcohol moderation:

Smoking cessation reduces vascular damage and improves BP outcomes. Moderate alcohol consumption, defined as ≤1 drink/day for women and ≤2 drinks/day for men, has been linked to improved cardiovascular health outcomes [58]. Alghamdi et al. (2023) provided insights into these behaviours and their impact on hypertension [59];

6. Stress management and yoga:

Yoga and mindfulness practices have gained recognition as adjunctive therapies. A study by Salagre et al. (2021) demonstrated significant reductions in BP among patients practising yoga regularly [60].

For patients who do not achieve adequate BP control through lifestyle modifications, pharmacological therapy is essential. Updated guidelines incorporate recent evidence to refine recommendations on medication classes and treatment strategies.

### 5.3. Pharmacological Management

#### 5.3.1. First-Line Antihypertensive Agents

The 2023 and 2024 guidelines recommend the following first-line agents (Table 2):

#### 5.3.2. Combination Therapy

The 2024 ESC guidelines recommend initiating combination therapy for most patients, particularly those with stage 2 hypertension or significantly elevated BP. A common combination is ACE inhibitors or ARBs with CCBs or thiazide diuretics. A study by Hasebe et al. (2020) in the Journal of Clinical Hypertension compared monotherapy with combination therapy involving amlodipine, irbesartan, and indapamide [61]. The study demonstrated that combination therapy significantly improved BP control and reduced cardiovascular events, supporting its use as a first-line treatment in patients with high-risk hypertension.

#### 5.3.3. Beta-Blockers

Beta-blockers remain crucial for patients with comorbidities such as CAD, heart failure, or AF. Although not first-line for hypertension alone, they are strongly indicated for these conditions. A meta-analysis by Kotecha et al. (2020) published in BMC Medicine assessed the efficacy of beta-blockers across various cardiovascular indications [62]. It demonstrated significant reductions in mortality, particularly in patients with heart failure and AF, reinforcing their critical role in managing HHD with these comorbidities. Beta-blockers are especially valuable in improving outcomes in AF by controlling heart rate and reducing thromboembolic risks when combined with anticoagulation therapy.

#### 5.3.4. Sodium–Glucose Co-Transporter-2 (SGLT2) Inhibitors

SGLT2 inhibitors have emerged as a game-changing therapy, particularly in patients with heart failure or CKD. A meta-analysis by Zelniker et al. (2019) in The Lancet showed that SGLT2 inhibitors reduced hospitalisations for heart failure by 23%. The benefits extended to non-diabetic populations, highlighting the broad applicability of this drug class in hypertension management [63]. SGLT2 inhibitors also provide renal protection, making them ideal for hypertensive patients at risk of CKD progression [64].

### 5.4. Management of Complications

#### 5.4.1. Heart Failure Management

For patients with HHD who develop heart failure, management is guided by heart failure-specific protocols, emphasising BP control and symptom relief. A recent study by Borlaug et al. (2024) in JAMA Cardiology highlights the significant prevalence of HFpEF in patients with severe secondary tricuspid regurgitation, underscoring the importance of early diagnosis and comprehensive management [65]. Evidence from the CVOT Summit Report (2023) in Cardiovascular Diabetology showed that SGLT2 inhibitors provide robust benefits in HFpEF, improving both cardiovascular and kidney outcomes, even in non-diabetic patients [66]. The ESC guidelines recommend diuretics for managing fluid overload, with strict sodium restriction to prevent volume expansion [2].

#### 5.4.2. AF Management

Patients with HHD are at high risk of developing AF, which increases stroke and heart failure risks. The guidelines recommend anticoagulation using direct oral anticoagulants (DOACs) or warfarin in eligible patients, alongside rate or rhythm control using beta-blockers or antiarrhythmic drugs. Catheter ablation may also be considered for refractory cases, as highlighted in a 2024 European Heart Rhythm Association/Heart Rhythm Society/Asia Pacific Heart Rhythm Society/Latin American Heart Rhythm Society consensus statement based on improved outcomes with this intervention in hypertensive patients with AF [67].

### 5.5. Interventional Therapies

The 2024 ESC guidelines introduce renal denervation as a validated option for resistant hypertension [2]. This minimally invasive procedure, which ablates sympathetic nerves in the renal arteries, has shown promising results in recent trials, lowering BP by an average of 10–12 mmHg. A 2023 study in the Journal of the American College of Cardiology confirmed its safety and efficacy in patients unresponsive to conventional therapy, marking a paradigm shift in managing resistant hypertension [68].

### 5.6. BP Targets in 2024 Guidelines

The 2024 guidelines advocate for tighter systolic BP targets of 120–129 mmHg for most patients, reflecting evidence that more intensive BP control reduces cardiovascular events (Table 3) [2]. However, these targets must be individualised, particularly in older or frail patients, where an ALARA (As Low As Reasonably Achievable) approach is recommended to balance benefits with potential adverse effects.

This comprehensive approach to HHD management integrates evidence-based recommendations and novel therapies to optimise outcomes for patients with this high-risk condition.

## 6. Special Populations and Complications

The management of HHD must be tailored to address the unique challenges and comorbidities faced by specific populations. Groups such as the elderly, patients with diabetes, and CKD, and those with AF require individualised strategies to achieve optimal BP control and prevent complications. The 2023 and 2024 guidelines emphasise the importance of tailoring treatment to these populations to mitigate risks of heart failure, arrhythmias, ischemic heart disease, and other complications.

### 6.1. Elderly and Frail Populations

In elderly and frail individuals, the management of hypertension presents unique challenges due to age-related arterial stiffness, altered drug metabolism, and a higher prevalence of comorbidities such as diabetes, CKD, and AF. For most elderly adults, the 2024 ESC guidelines recommend a systolic BP target of 120–129 mmHg [2]. However, for frail individuals, a more cautious approach is advised, emphasising ALARA targets to balance the benefits of BP reduction with the risks of hypotension, falls, and cognitive impairment. For patients over 85 years old, a systolic BP target of less than 140 mmHg is often deemed appropriate, particularly in those with a limited life expectancy or risk of orthostatic hypotension.

Elderly patients with HHD are at higher risk of complications, including HFpEF and AF. Age-related diastolic dysfunction and LVH contribute to the prevalence of HFpEF in this population, while atrial dilation and chronic hypertension increase the incidence of AF. Effective BP control reduces the risk of these complications, but the choice of medications must consider potential adverse effects such as dehydration from diuretics or bradycardia induced by beta-blockers.

### 6.2. Patients with Diabetes

Patients with type 2 diabetes are at an elevated risk for hypertensive complications due to the synergistic effects of diabetes and hypertension on the cardiovascular system. Diabetes accelerates the progression of atherosclerosis, myocardial fibrosis, and CKD, increasing the likelihood of myocardial infarction, stroke, heart failure, and sudden cardiac death.

The 2023 and 2024 guidelines advocate for aggressive BP management in patients with diabetes, recommending systolic BP reduction to 120–129 mmHg to lower cardiovascular risks and slow renal disease progression [2,7]. RAAS inhibitors, such as ACE inhibitors or ARBs, are the first-line agents in this population, owing to their ability to reduce albuminuria and preserve kidney function.

Diabetic patients with HHD are more likely to develop ischemic heart disease due to accelerated atherosclerosis. They are also at increased risk for heart failure, particularly HFpEF, as well as arrhythmias such as AF. Managing these complications often necessitates the use of glucose-lowering agents with cardiovascular benefits, such as SGLT2 inhibitors, alongside traditional antihypertensive therapies.

### 6.3. Patients with CKD

Hypertension and CKD frequently coexist, with each condition exacerbating the other. Hypertension accelerates CKD progression, while CKD worsens hypertension through mechanisms such as sodium retention and increased RAAS activity. The 2024 guidelines emphasise the importance of stringent BP control in these patients, recommending a systolic BP target of less than 130 mmHg, ideally within a range of 120–129 mmHg if tolerable [2]. RAAS inhibitors, particularly ACE inhibitors or ARBs, are the preferred first-line agents due to their demonstrated efficacy in reducing proteinuria and slowing renal decline.

CKD patients with hypertension face an increased risk of heart failure, particularly HFpEF, due to the combined effects of volume overload, myocardial fibrosis, and LVH. Arrhythmias, including AF, are also more common in this population, compounding their risk of stroke and worsening heart failure. Careful titration of medications is essential to avoid electrolyte imbalances and further renal damage, with diuretics frequently used to manage fluid overload.

### 6.4. Patients with AF

AF is a common complication in patients with long-standing hypertension, often resulting from left atrial remodelling and LVH. AF increases the risk of thromboembolic events, including stroke, and exacerbates heart failure. Its presence complicates the management of hypertension, requiring concurrent strategies for BP control and stroke prevention.

The 2023 and 2024 guidelines recommend DOACs over warfarin for stroke prevention in hypertensive patients with AF, given their superior efficacy and safety [2,7]. Rate control is often achieved with beta-blockers or non-dihydropyridine calcium channel blockers, while rhythm control may involve antiarrhythmic drugs such as amiodarone or catheter ablation in selected cases.

Optimal BP control is critical in hypertensive patients with AF, as poorly controlled hypertension exacerbates atrial remodelling and increases the frequency of recurrent AF episodes. The 2024 ESC guidelines recommend a systolic BP target of 120–129 mmHg in this population, as achieving this target reduces the risk of stroke and heart failure while improving overall cardiovascular outcomes [2].

## 7. Conclusions

The management of HHD has evolved substantially with the release of the 2023 and 2024 hypertension guidelines. These updates emphasise a more aggressive approach to BP control, recommending a systolic BP target of 120–129 mmHg for most patients, a significant departure from earlier guidelines that suggested more conservative thresholds. The introduction of the “elevated BP” category (120–139/70–89 mmHg) encourages earlier intervention, particularly in patients with additional cardiovascular risk factors such as diabetes or CKD.

Incorporating renal denervation as a therapeutic option for resistant hypertension represents a major advancement in non-pharmacological treatment, providing clinicians with a new tool for patients who are unresponsive to traditional therapies. Additionally, the guidelines underscore the importance of managing comorbidities, such as heart failure and AF, alongside hypertension, with tailored approaches for high-risk groups like the elderly, diabetics, and those with CKD.

The shift toward more intensive BP targets, combined with personalised management strategies based on cardiovascular risk stratification, marks a transformative era in HHD care. These evidence-based updates provide clinicians with a comprehensive framework to improve patient outcomes, prevent disease progression, and reduce cardiovascular events. Adopting these guidelines in clinical practice will be critical in addressing the global burden of HHD and optimising care for at-risk populations.

## Figures and Tables

**Table 1:** Comparison of risk stratification criteria in ESC vs. ESH Guidelines. ESC, European Society of Cardiology; ESH, European Society of Hypertension; HMOD, hypertension-mediated organ damage.

Criteria	2018 Guidelines	ESH 2023	ESC 2024
Risk Score Algorithm	Score (older version of SCORE2)	SCORE2 (10-year cardiovascular risk)	SCORE2
Regional Variations	No	No	Yes, across Europe
Inclusion of HMOD	Moderate emphasis	Moderate emphasis, traditional techniques	Strong emphasis, with advanced imaging
BP Target for High Risk	<140 mmHg	<130 mmHg	<120–129 mmHg

**Table 2:** First-line anti-hypertensive agents recommended in the European Society of Hypertension 2023 and European Society of Cardiology 2024 guidelines for the management of hypertension [2,7]. ACE, angiotensin-converting enzyme; ARB, angiotensin receptor blocker; BP, blood pressure; CCB, calcium channel blocker; LVH, left ventricular hypertrophy.

Medications	Description
ACE Inhibitors and ARBs	Widely used for their ability to reduce myocardial remodelling, improve left ventricular function, and lower BP. Particularly effective in patients with LVH or high cardiovascular risk.
CCBs	Effective at lowering BP by reducing systemic vascular resistance. Dihydropyridines (e.g., amlodipine) are preferred in patients with angina or coronary artery disease.
Thiazide and Thiazide-like Diuretics	Drugs, such as chlorthalidone, reduce BP by promoting sodium excretion.

**Table 3:** Comparison of BP targets in ESC and ESH guidelines, including targets for special populations. ESC, European Society of Cardiology; ESH, European Society of Hypertension; CVD, cardiovascular disease; CKD, chronic kidney disease.

Population	ESC 2018	ESC 2024	ESH 2018	ESH 2023
General population	<140/90 mmHg	120–129 mmHg	<140/90 mmHg	<130 mmHg
High risk (CVD/CKD/Diabetes)	<140 mmHg	<120–129 mmHg	<140/90 mmHg	<130 mmHg
Older adults (>65 years)	<140–150 mmHg	<140 mmHg	<140–150 mmHg	<140 mmHg

## Data Availability

No original data are to be made available.

## Abbreviations

ABPM	Ambulatory blood pressure monitoring
ACC	American College of Cardiology
ACE	Angiotensin-converting enzyme
AF	Atrial fibrillation
AHA	American Herat Association
ALARA	As Low As Reasonably Achievable
ARB	Angiotensin receptor blocker
BNP	B-type natriuretic peptide
BP	Blood pressure
CAD	Coronary artery disease
CKD	Chronic kidney disease
CVD	Cardiovascular disease
DASH	Dietary Approaches to Stop Hypertension
DOA	Direct oral anticoagulant
ECG	Electrocardiography
ESC	European Society of Cardiology
ESH	European Society of Hypertension
ESRD	End-stage renal disease
HBPM	Home blood pressure monitoring
HHD	Hypertensive heart disease
HFpEF	Heart failure with preserved ejection fraction
HFrEF	Heart failure with reduced ejection fraction
LVH	Left ventricular hypertrophy
MRI	Magnetic resonance imaging
RAAS	Renin–angiotensin–aldosterone system
SGLT	Sodium–Glucose Co-Transporter-2
STEP	Systolic Blood Pressure Intervention Trial
STEP	Strategy of Blood Pressure Intervention in the Elderly Hypertensive Patients

## References

[1] Whelton P.K., Carey R.M., Mancia G., Kreutz R., Bundy J. (2022). Harmonization of the American College of Cardiology/American Heart Association and European Society of Cardiology/European Society of Hypertension Blood Pressure/Hypertension Guidelines: Comparisons, Reflections, and Recommendations. Circulation.

[2] McEvoy J.W., McCarthy C.P., Bruno R.M., Brouwers S., Canvan M., Ceconi C., Christodorescu R., Daskalopoulou S., Ferro C., Gerdts E. (2024). 2024 ESC Guidelines for the management of elevated blood pressure and hypertension. Eur. Heart J..

[3] Williams B., Mancia G., Spiering W., Rosei E., Azizi M., Burnier M., Clement D., Coca A., De Simone G., Dominiczak A. (2018). 2018 ESC/ESH Guidelines for themanagement of arterial hypertension. Eur. Heart J..

[4] Levine G.N., Al-Khatib S.M., Beckman J.A., Birtcher K., Bozkurt B., Brindis R., Cigarroa J., Curtis L., Deswal A., Fleisher L. (2018). Force on Clinical Practice Guidelines. Hypertension.

[5] SPRINT Research Group (2015). A Randomized Trial of Intensive versus Standard Blood-Pressure Control. N. Engl. J. Med..

[6] Zhang W., Zhang S., Deng Y., Wu S., Ren J., Sun G., Yang J., Jiang Y., Xu X., Wang T. (2021). Trial of Intensive Blood-Pressure Control in Older Patients with Hypertension. New Engl. J. Med..

[7] Mancia G., Kreutz R., Brunstr M., Burnier M., Grassi G., Januszewicz A., Lorenza Muiesan M., Tsioufis K., Agabiti-Rosei E., Abd Elhady Algharably E. (2023). ESH Guidelines. https://journals.lww.com/jhypertension.

[8] Carey R.M., Calhoun D.A., Bakris G.L., Brook R., Daugherty S., Dennison-Himmelfarb C., Egan B., Flack J., Gidding S., Judd E. (2018). Resistant hypertension: Detection, evaluation, and management a scientific statement from the American Heart Association. Hypertension.

[9] Messerli F.H., Bangalore S. (2013). Treatment-resistant hypertension: Another Cinderella story. Eur. Heart J..

[10] Saheera S., Krishnamurthy P. (2020). Cardiovascular Changes Associated with Hypertensive Heart Disease and Aging. Cell Transplant..

[11] Masenga S.K., Kirabo A. (2023). Hypertensive heart disease: Risk factors, complications and mechanisms. Front. Cardiovasc. Med..

[12] Martin T.G., Juarros M.A., Leinwand L.A. (2023). Regression of cardiac hypertrophy in health and disease: Mechanisms and therapeutic potential. Nat. Rev. Cardiol..

[13] Sayin B.Y., Oto A. (2022). Left Ventricular Hypertrophy: Etiology-Based Therapeutic Options. Cardiol. Ther..

[14] Lorell B.H., Carabello B.A. (2000). Left Ventricular Hypertrophy Pathogenesis, Detection, and Prognosis. https://www.ahajournals.org/doi/10.1161/01.CIR.102.4.470.

[15] Humphrey J.D. (2021). Mechanisms of vascular remodeling in hypertension. Am. J. Hypertens..

[16] Chirinos J.A., Segers P., Hughes T., Townsend R. (2019). Large-Artery Stiffness in Health and Disease: JACC State-of-the-Art Review. J. Am. Coll. Cardiol..

[17] Gallo G., Volpe M., Savoia C. (2022). Endothelial Dysfunction in Hypertension: Current Concepts and Clinical Implications. Front. Med..

[18] Kong P., Christia P., Frangogiannis N.G. (2014). The pathogenesis of cardiac fibrosis. Cell. Mol. Life Sci..

[19] Bunda S., Liu P., Wang Y., Hinek A. (2007). Aldosterone induces elastin production in cardiac fibroblasts through activation of insulin-like growth factor-I receptors in a mineralocorticoid receptor-independent manner. Am. J. Pathol..

[20] Messerli F.H., Rimoldi S.F., Bangalore S. (2017). Mini-Focus Issue: Cardiovascular Comorbidities The Transition From Hypertension to Heart Failure Contemporary Update. https://www.acc.org/jacc-journals-cme.

[21] Ekström M., Hellman A., Hasselström J., Hage C., Kahan T., Ugander M., Wallén H., Persson H., Linde C. (2020). The transition from hypertension to hypertensive heart disease and heart failure: The PREFERS Hypertension study. ESC Heart Fail.

[22] Vaidya K., Semsarian C., Chan K.H. (2017). Atrial Fibrillation in Hypertrophic Cardiomyopathy. Heart Lung Circ..

[23] Ames M.K., Atkins C.E., Pitt B. (2019). The renin-angiotensin-aldosterone system and its suppression. J. Vet. Intern. Med..

[24] Pisano A., Iannone L.F., Leo A. (2021). Russo, E.; Coppolino, G.; Bilignano, D. Renal denervation for resistant hypertension. Cochrane Database Syst. Rev..

[25] Hartupee J., Mann D.L. (2016). Neurohormonal activation in heart failure with reduced ejection fraction. Nat. Rev. Cardiol..

[26] Borovac J.A., D’Amario D., Bozic J., Glavas D. (2020). Sympathetic nervous system activation and heart failure: Current state of evidence and the pathophysiology in the light of novel biomarkers. World J. Cardiol..

[27] Maron M.S., Maron B.J., Harrigan C., Buros J., Gibson C., Olivotto I., Biller L., Lesser J., Udelson J., Manning W. (2009). Hypertrophic Cardiomyopathy Phenotype Revisited After 50 Years With Cardiovascular Magnetic Resonance. J. Am. Coll. Cardiol..

[28] Aronow W.S. (2017). Hypertension associated with atrial fibrillation. Ann. Transl. Med..

[29] Verdecchia P., Angeli F., Reboldi G. (2018). Hypertension and atrial fibrillation: Doubts and certainties from basic and clinical studies. Circ. Res..

[30] Nagueh S.F. (2020). Left Ventricular Diastolic Function: Understanding Pathophysiology, Diagnosis, and Prognosis With Echocardiography. JACC Cardiovasc. Imaging.

[31] Trivedi S.J., Altman M., Stanton T., Thomas L. (2019). Echocardiographic Strain in Clinical Practice. Heart Lung Circ..

[32] Bayram N., Akoğlu H., Sanri E., Karacabey S., Efeoglu M., Onur O., Denizbasi A. (2021). Diagnostic Accuracy of the Electrocardiography Criteria for Left Ventricular Hypertrophy (Cornell Voltage Criteria, Sokolow-Lyon Index, Romhilt-Estes, and Peguero-Lo Presti Criteria) Compared to Transthoracic Echocardiography. Cureus.

[33] Karamitsos T.D., Arvanitaki A., Karvounis H., Neubauer S., Ferreira V. (2020). Myocardial Tissue Characterization and Fibrosis by Imaging. JACC: Cardiovasc. Imaging.

[34] Grewal J., McKelvie R., Lonn E., Tait P., Carlsson J., Gianni M., Jarnert C., Persson H. (2008). BNP and NT-proBNP predict echocardiographic severity of diastolic dysfunction. Eur. J. Heart Fail.

[35] Lawson C.A., Zaccardi F., Squire I., Okhai H., Davies M., Huang W., Mamas M., Lam C.S., Khunti K., Kadam U.T. (2020). Risk Factors for Heart Failure: 20-Year Population-Based Trends by Sex, Socioeconomic Status, and Ethnicity. Circ. Heart Fail..

[36] Roumie C.L., Hung A.M., Russell G.B., Basile J., Kreider K., Nord J., Ramsey T., Rastogi A., Sweeney M., Tamariz L. (2020). Blood Pressure Control and the Association with Diabetes Mellitus Incidence: Results from SPRINT Randomized Trial. Hypertension.

[37] Tokgozoglu L., Torp-Pedersen C. (2021). Redefining cardiovascular risk prediction: Is the crystal ball clearer now?. Eur. Heart J..

[38] Goff D.C., Lloyd-Jones D.M., Bennett G., Coady S., D’Agostino R., Gibbons R., Greenland P., Lackland D., Levy D., O’Donnell C. (2014). 2013 ACC/AHA guideline on the assessment of cardiovascular risk: A report of the American college of cardiology/American heart association task force on practice guidelines. Circulation.

[39] Jenkins S., Cross A., Osman H., Salim F., Lane D., Bernieh D., Khunti K., Gupta P. (2024). Effectiveness of biofeedback on blood pressure in patients with hypertension: Systematic review and meta-analysis. J. Hum. Hypertens..

[40] Ettehad D., Emdin C.A., Kiran A., Anderson S., Callender T., Emberson J., Chalmers J., Rodgers A., Rahimi K. (2016). Blood pressure lowering for prevention of cardiovascular disease and death: A systematic review and meta-analysis. Lancet.

[41] Reinier K., Dervan C., Singh T., Uy-Evanado A., Lai S., Gunson K., Jui J., Chugh S. (2011). Increased left ventricular mass and decreased left ventricular systolic function have independent pathways to ventricular arrhythmogenesis in coronary artery disease. Heart Rhythm..

[42] Haider A.W., Larson M.G., Benjamin E.J., Levy D. (1998). Increased Left Ventricular Mass and Hypertrophy Are Associated With Increased Risk for Sudden Death. J. Am. Coll. Cardiol..

[43] Bing R., Dweck M.R. (2019). Myocardial fibrosis: Why image, how to image and clinical implications. Heart.

[44] Zhan Q., Peng W., Wang S., Gao J. (2023). Heart Failure with Preserved Ejection Fraction: Pathogenesis, Diagnosis, Exercise, and Medical Therapies. J. Cardiovasc. Transl. Res..

[45] Liang M., Bian B., Yang Q. (2022). Characteristics and long-term prognosis of patients with reduced, mid-range, and preserved ejection fraction: A systemic review and meta-analysis. Clin. Cardiol..

[46] Miró Ò., Conde-Martel A., Llorens P., Salamanca-Bautista P., Gil V., González-Franco Á., Jacob J., Casado J., Tost J., Montero-Perez-Barquero M. (2023). The influence of comorbidities on the prognosis after an acute heart failure decompensation and differences according to ejection fraction: Results from the EAHFE and RICA registries. Eur. J. Intern. Med..

[47] Papademetriou V., Zaheer M., Doumas M., Lovato L., Applegate W., Tsioufis C., Mottle A., Punthakee Z., Cushman M. (2016). Cardiovascular outcomes in action to control cardiovascular risk in diabetes: Impact of blood pressure level and presence of kidney disease. Am. J. Nephrol..

[48] Izraiq M., Alawaisheh R., Ibdah R., Dabbas A., Ahmed Y., Mughrabi Sabbagh A., Zuriak A., Ababneh M., Toubasi A., Al-Bkoor B. (2024). Machine Learning-Based Mortality Prediction in Chronic Kidney Disease among Heart Failure Patients: Insights and Outcomes from the Jordanian Heart Failure Registry. Medicina (Lithuania).

[49] Hagi K., Kochi K., Watada H., Haku K., Ueki K. (2023). Effect of patient characteristics on the efficacy and safety of imeglimin monotherapy in Japanese patients with type 2 diabetes mellitus: A post-hoc analysis of two randomized, placebo-controlled trials. J. Diabetes Investig..

[50] Campbell-Quintero S., Echeverría L.E., Gómez-Mesa J.E., Rivera-Toquica A., Renteria-Asprilla C., Lopez-Garzon N., Alcala-Hernandez A., Accini-Mendoza J., BAquero-Lozano G., Martinez-Carvajal A. (2023). Comorbidity profile and outcomes in patients with chronic heart failure in a Latin American country: Insights from the Colombian heart failure registry (RECOLFACA). Int. J. Cardiol..

[51] Maniero C., Lopuszko A., Papalois K.B., Gupta A., Kapil A., Khanji M. (2023). Non-pharmacological factors for hypertension management: A systematic review of international guidelines. Eur. J. Prev. Cardiol..

[52] Whelton P.K., Carey R.M., Aronow W.S., Casey D., Collins K., Dennison Himmelfarb C., DePalma S., Gidding S., Jamerson K., Jones D. (2018). 2017 ACC/AHA/AAPA/ABC/ACPM/AGS/APhA/ASH/ASPC/NMA/PCNA Guideline for the Prevention, Detection, Evaluation, and Management of High Blood Pressure in Adults: Executive Summary: A Report of the American College of Cardiology/American Heart Association Task Force on Clinical Practice Guidelines. Circulation.

[53] Khani Jeihooni A., Sobhani A., Afzali Harsini P., Amirkhani M. (2023). Effect of educational intervention based on PRECEDE model on lifestyle modification, self-management behaviors, and hypertension in diabetic patients. BMC Endocr. Disord..

[54] Rossi A.M., Moullec G., Lavoie K.L., Gour-Provençal G., Bacon S. (2013). The evolution of a Canadian hypertension education program recommendation: The impact of resistance training on resting blood pressure in adults as an example. Can. J. Cardiol..

[55] Pescatello L.S., Franklin B.A., Fagard R., Farquhar W.B., Kelley G.A., Ray C.A. (2004). Exercise and Hypertension. Med. Sci. Sports Exerc..

[56] Tsoi K., Lam A., Tran J., Hao Z., Yiu K., Chia Y., Turana Y., Siddique S., Zhang Y., Cheng H. (2023). The Western and Chinese exercise training for blood pressure reduction among hypertensive patients: An overview of systematic reviews. J. Clin. Hypertens..

[57] Kodela P., Okeke M., Guntuku S., Lingamsetty S., Slonovschi E. (2023). Management of Hypertension With Non-pharmacological Interventions: A Narrative Review. Cureus.

[58] O’Keefe E.L., DiNicolantonio J.J., O’Keefe J.H., Lavie C. (2018). Alcohol and CV Health: Jekyll and Hyde J-Curves. Prog. Cardiovasc. Dis..

[59] Ballut O.M., Alzahrani A.A., Alzahrani R.A., Alzahrani A.T., Alzahrani R.A., Alzahrani M.F., Alzahrani Y.K., Alghamdi N., Alghamdi R. (2023). The Impact of Non-pharmacological Interventions on Blood Pressure Control in Patients With Hypertension: A Systematic Review. Cureus.

[60] Hadaye R., Shastri S., Salagre S. (2021). Effect of yoga intervention in the management of hypertension: A preventive trial. Int. J. Prev. Med..

[61] Nakagawa N., Sato N., Saijo Y., Morimoto H., Koyama S., Ogawa Y., Uekita K., Maruyama J., Ohta T., Nakamura Y. (2020). Assessment of suitable antihypertensive therapies: Combination with high-dose amlodipine/irbesartan vs triple combination with amlodipine/irbesartan/indapamide (ASAHI-AI study). J. Clin. Hypertens..

[62] Ziff O.J., Samra M., Howard J.P., Bromage D., Rhuschitzka F., Francis D., Kotecha D. (2020). Beta-blocker efficacy across different cardiovascular indications: An umbrella review and meta-analytic assessment. BMC Med..

[63] Zelniker T.A., Wiviott S.D., Raz I., Im K., Goodrich E., Bonaca M., Mosenzon O., Kato E., Cahn A., Furtado R. (2019). SGLT2 inhibitors for primary and secondary prevention of cardiovascular and renal outcomes in type 2 diabetes: A systematic review and meta-analysis of cardiovascular outcome trials. Lancet.

[64] Mende C.W. (2022). Chronic Kidney Disease and SGLT2 Inhibitors: A Review of the Evolving Treatment Landscape. Adv. Ther..

[65] Naser J.A., Harada T., Reddy Y.N., Pislaru S., Michelena H., Scott C., Kennedy A., Pellikka P., Nkomo V., Eleid M. (2024). Prevalence of HFpEF in Isolated Severe Secondary Tricuspid Regurgitation. JAMA Cardiol..

[66] Schnell O., Barnard-Kelly K., Battelino T., Ceriello A., Larsson H., Fernandez-Fernandez B., Forst T., Frias J., Gavin J., Giorgino F. (2024). CVOT Summit Report 2023, new cardiovascular, kidney, and metabolic outcomes. Cardiovasc. Diabetol..

[67] Tzeis S., Gerstenfeld E.P., Kalman J., Saad E., Seperhi Shamloo A., Andrade J., Barbhaiya C., Baykaner T., Boveda S., Calkins H. (2024). 2024 European Heart Rhythm Association/Heart Rhythm Society/Asia Pacific Heart Rhythm Society/Latin American Heart Rhythm Society expert consensus statement on catheter and surgical ablation of atrial fibrillation. Europace.

[68] Kandzari D.E., Townsend R.R., Kario K., Mahfoud F., Weber M., Schmeider R., Pocock S., Tsioufis K., Konstantinidis D., Choi J. (2023). Safety and Efficacy of Renal Denervation in Patients Taking Antihypertensive Medications. J. Am. Coll. Cardiol..

